# Comparing hypofractionated to conventional fractionated radiotherapy in postmastectomy breast cancer: a meta-analysis and systematic review

**DOI:** 10.1186/s13014-020-1463-1

**Published:** 2020-01-17

**Authors:** Lei Liu, Yongqiang Yang, Qi Guo, Bixin Ren, Qiliang Peng, Li Zou, Yaqun Zhu, Ye Tian

**Affiliations:** Department of Radiotherapy and Oncology, The Second Affiliated Hospital of Soochow University, Institute of Radiotherapy and Oncology of Soochow University, San Xiang Road No. 1055, Suzhou, 215004 Jiangsu China

**Keywords:** Breast cancer, Hypofractionated radiotherapy, Conventional radiotherapy, Postmastectomy

## Abstract

**Purpose:**

To compare the efficacy and toxicity of hypofractionated radiotherapy versus conventional fractionated radiotherapy in postmastectomy breast cancer using meta-analysis.

**Methods:**

The PubMed, EMbase, Cochrane Library, Google Scholar, Wan Fang and CNKI databases were searched to identify controlled clinical trials comparing hypofractionated radiotherapy versus conventional fractionated radiotherapy in postmastectomy breast cancer. Overall survival (OS) was the primary endpoint, and disease-free survival (DFS), locoregional recurrence (LRR), distant metastasis (DM), acute skin toxicity, acute lung toxicity, late skin toxicity, lymphedema,, shoulder restriction, and late cardiac related toxicity were the secondary endpoints.

**Results:**

Twenty-five controlled clinical trials involving 3871 postmastectomy breast cancer patients were included in this meta-analysis according to the selection criteria. The meta-analysis revealed that there were no significant differences in OS (*OR* = 1.08, 95% *CI* = 0.87~1.33, *P* = 0.49), DFS (*OR* = 1.13, 95% *CI* = 0.91~1.40, *P* = 0.28), LRR (*OR* = 1.01, 95% *CI* = 0.76~1.33, *P* = 0.96), DM (*OR* = 1.16, 95% *CI* = 0.85~1.58, *P* = 0.34), acute skin toxicity (*OR* = 0.94, 95% *CI* = 0.67~1.32, *P* = 0.72), acute lung toxicity (*OR* = 0.94, 95% *CI* = 0.74~1.20, *P* = 0.62), late skin toxicity (*OR* = 0.98, 95% *CI* = 0.75~1.27, *P* = 0.88), lymphedema (*OR* = 0.99, 95% *CI* = 0.77~1.28, *P* = 0.94), shoulder restriction (*OR* = 0.75, 95% *CI* = 0.43~1.31, *P* = 0.31), or late cardiac related toxicity (*OR* = 1.17, 95% *CI* = 0.82~1.65, *P* = 0.39) between the two groups.

**Conclusions:**

The results of this study show that compared to conventional fractionated radiotherapy, hypofractionated radiotherapy is not significantly different with respect to efficacy or toxicity in postmastectomy breast cancer. Additional large randomized clinical trials are needed to further confirm this conclusion.

## Introduction

Breast cancer has the highest incidence rate and causes the second highest number of deaths among cancers in women according to cancer statistics from 2019 in the United States [1]. It is well accepted that postmastectomy radiotherapy (PMRT) improves long-term outcomes by reducing local recurrence and cancer mortality in breast cancer after mastectomy [2, 3]. The most recent National Comprehensive Cancer Network (NCCN) guidelines recommend the conventional fractionated radiotherapy (CFRT) schedule for PMRT, which consists of a total dose (TD) of 45.0 Gy to 50.4 Gy given in 25 to 28 fractions over 5 weeks or more and delivered to the chest wall and regional lymph nodes. However, with advances in radiotherapy technology, methods to reduce toxicity, overall treatment time and cost have gradually attracted the attention of researchers.

Previous reports indicate that breast cancer has a low ratio of α/β over the range of 2.0~4.0 Gy, and this low α/β ratio suggests that the efficacy of hypofractionated radiotherapy (HFRT) regimens are equivalent to CFRT in breast cancer [4]. Data from randomized controlled trials from the United Kingdom and Canada confirm this conclusion to a certain extent [5–11]. However, most of the patients in these trials were early breast cancer patients who underwent breast-conserving surgery. Adjuvant treatment of breast cancer after mastectomy remains controversial, and there are few relevant prospective randomized controlled clinical trials (RCTs) that address adjuvant treatment internationally. In *Lancet Oncology, 2019*, Wang et al. [12] report 5-year outcomes of a randomized, non-inferiority, open-label, phase 3 trial in China that compared postmastectomy HFRT with CFRT directed to the chest wall and the supraclavicular and level III axillary nodal regions in 820 patients with locally advanced breast cancer (at least four positive axillary lymph nodes). There were no significant differences in the 5-year cumulative incidence of locoregional recurrence, 5-year overall survival or 5-year disease-free survival between groups. Furthermore, acute and late toxicities were similar in both groups. This finding suggests that hypofractionated postmastectomy radiotherapy (HF PMRT) is safe and effective for patients with a high risk for breast cancer, exhibiting low toxicity and high local control rates. In addition, 15% (336/2236) and 8% (177/2215) of patients with postmastectomy HFRT were included in the START A and START B trials, respectively, and there was no significant difference in local recurrence or late toxicities between the two groups over a long-term follow-up of 10 years [5–8].

Clinically, HFRT could reduce the cost of cancer treatment, provide more convenient treatment and allow providers to treat more patients. There is growing interest in using the HF PMRT scheme, although the number of patients receiving HFRT after mastectomy in the United States is currently small (1.1%) [13]. In recent years, HFRT use in patients who underwent breast-conserving surgery has been written into NCCN and other treatment guidelines and has been gradually applied in the clinic, but using HFRT in patients after mastectomy remains controversial. Therefore, we aimed to use evidence-based medicine to compare the efficacy and toxicity of HFRT and CFRT after mastectomy.

## Methods

### Study protocol

A search of PubMed, EMBASE, Cochrane Library, Google Scholar, Wan Fang, and CNKI was conducted up through February 25, 2019. MeSH or Emtree terms combined free terms were used: “breast cancer”, “mastectomy”, “radiotherapy”, “hypofractionated” and “conventional fractionated”.

### Selection criteria

Inclusion Criteria: (1) Surgical mastectomy in patients diagnosed with breast cancer by pathology. (2) Controlled trials comparing HFRT to CFRT after mastectomy. (3) Inclusion of study sample size > 20 cases. (4) Complete information is provided in the literature. Exclusion Criteria: (1) Review articles, case reports, meeting abstracts, and lectures. (2) No clear diagnosis was made in enrolled patients. (3) Inclusion of study sample size < 20 cases. (4) Incorrect data, incomplete data or unable to extract required data. (5) Duplicate publications.

### Data extraction

Data were independently screened and extracted by two reviewers, including patient eligibility, study design, baseline characteristics, and number of events for all outcomes and interventions. Overall survival (OS) was the primary endpoint, and disease-free survival (DFS), locoregional recurrence (LRR), distant metastasis (DM), acute skin toxicity, acute lung toxicity, late skin toxicity, lymphedema, shoulder restriction, and late cardiac related toxicity were secondary endpoints. Any disagreement was resolved by consensus.

### Quality assessment

Quality assessment of included studies was independently performed by two authors, and disagreements were resolved by consensus. Study quality was evaluated using the Newcastle-Ottawa scale (NOS). Primary evaluations included measurement of exposure factors, comparability between groups, and patient selection. Each study with NOS scores ≥6 was considered a high-quality study, whereas studies with NOS scores<6 were considered low-quality studies. Quality assessment results of included studies are summarized in Table 1.

**Table 1:** Baseline characteristics and dose fraction of 3871 patients in 25 studies with breast cancer after mastectomy

Study (year published)	Sample size		Age (years)	Clinical stage	Interventions (Gy/fractions)		Outcomes	NOS	The type of RT technique	RT area and prescriptive method used	Bolus	Breast reconstruction
	HFRT	CFRT			HFRT	CFRT						
Wang (2019) [12]	401	409	24~74	II~III	43.5/15	50/25	OS, DFS, LRR, Toxicities	9	2DRT, 3DCRT, or IMRT	CW: a single appositional field with 6–9 MeV electron beam, depending on CW thickness SPC: anteroposterior field using a 6 MV photon, combined 6 MV photon, and 9–15 MeV electron or a 10~12 MeV electron beam	5 mm bolus to CW	None
Abhilash (2016) [14]	30	30	25~67	II~III	39/13	50/25	LRR, Toxicities	6	2DRT	CW: bilateral tangent pair fields SPC: a direct anterior field Cobalt 60 teletherapy machine with gamma energy 1.25 MeV	NR	None
Bi (2011) [15]	51	49	28~64	II~III	43.5/15	50/25	OS, LRR, Toxicities	6	2DRT	CW: a 6~12 MeV electron beam SPC: 12 MeV electron and 6 MV X-ray beams mixed	5 mm bolus to CW	None
Bedi (2018) [16]	30	30	39~61	II~III	42.5/16	50/25	Toxicities	5	3DCRT	CW: two tangential fields using 6 MV X-ray beam SPC: a direct anterior field with 6 MV photon beam	Universal bolus to CW	None
Das (2018) [17]	55	53	48/50 ^a^	I~III	42.56/16	50/25	DFS, LRR, DM, Toxicities	6	2DRT	CW: medial and lateral tangential fields SPC: anteroposterior field Cobalt 60 teletherapy machine	NR	None
Eldeeb (2012) [18]	66	41	25~67	I~III	45/17、40/15	50/25	LRR, Toxicities	7	2DRT	CW: two tangential fields SPC and Axillary: an anterior field 6 MV photon linear accelerator or Cobalt 60 machine	NR	None
El-Sayed (2012) [19]	159	84	30~69	II~III	42.5/16、39/13	50/25	OS, DFS, LRR, Toxicities	7	2DRT	CW: medial and lateral tangential fields using 6 MV photon beam SPC: anteroposterior field using 6 MV photon beam	NR	None
Elsayed (2014) [20]	25	22	33~70	II~III	42.72/16	50/25	OS, DFS, Toxicities	8	2DRT	CW and Axillary: two tangential fields using 6 MV photon beam SPC: a direct anterior field using 6 MV photon beam	NR	None
Fatma (2018) [21]	50	50	31~68	II~III	40/15	50/25	DFS, LRR, Toxicities	7	3DCRT	CW: two tangential fields using 6 MV or 15 MV photon beam SPC: an anterior field using 6 MV or 15 MV photon beam	NR	None
He (2016) [22]	48	63	25~57	I~II	40/15	50/25	Toxicities	6	3DCRT	CW and SPC: 6 MV photon beam	NR	None
Huang (2013) [23]	70	70	25~65	II~III	43.5/15	48.5/22	LRR	6	NR	RT area: CW and SPC	NR	None
Jin (2013) [24]	164	136	23~75	NR	45/15	50/25	OS, DM, LRR, Toxicities	6	NR	RT area: CW, SPC, Axillary, Axillary dome or internal mammary lymph node region if necessary	NR	None
Kalita (2018) [25]	25	25	27~70	II~III	40/15	50/25	Toxicities	5	2DRT,	CW: two parallel opposed tangential fields SPC and Axillary: a separate direct anterior field All fields using 6 MV photon beam	NR	None
Kouloulias (2016) [26]	87	30	33~78	II~III	48.3/21、42.56/16	50/25	Toxicities	7	3DCRT	CW and SPC using 6 MV photon beam	NR	None
Kumbhaj (2013) [27]	46	45	31~70	I~III	40/17	50/25	DFS, LRR, DM, Toxicities	6	2DRT,	CW: tangent pair technique by using Cobalt 60 machine; SPC: NR	NR	None
Pinitpatcharalert (2011) [28]	148	67	44~56	I~III	42.72/16	50/25	OS, DFS, Toxicities	8	2DRT	CW: medial and lateral tangential chest wall fields SPC: anteroposterior ipsilateral supraclavicular field Axillary boost: clinical N2 disease, inadequate node excision (less than 10 nodes), or perinodal invasion CW and SPC field were treated with cobalt 60	5 mm bolus to CW	None
Purohit (2016) [29]	25	25	18~65	II~III	40/15	50/25	Toxicities	6	2DRT	CW: medial tangential and lateral tangential fields SPC: anteroposterior field two field technique by using Cobalt 60 energy source	NR	None
Rastogi (2018) [30]	50	50	21~79	II~III	42.72/16	50/25	DFS, LRR, DM	7	3DCRT	CW and SPC: single beam energy, or a combination of both 6 and 15 MV photon beam was used depending on patients’ anatomy		None
Wang (2010) [31]	31	30	32~71	NR	41.6/13	50/25	OS, Toxicities	5	2DRT	CW and SPC: 12 MeV electron and 6 MV X-ray beams mixed, two parallel opposed tangential fields	NR	None
Wu (2003) [32]	177	149	26~74	I~III	45/15	50/25	OS, LRR	8	2DRT,	SPC, Axillary and Axillary dome: 8 MV X-ray, and the 20 Gy SPC was added with 1.5 cm bolus Internal mammary: 14 MeV electron beam or mixed with 8 MV X-ray; CW: tangent to 8 MV X-ray or 6–8 MeV electron beam	1.5 cm bolus to SPC	None
Wu (2014) [33]	53	53	31~68	II~IV ^b^	42/15	50/25	NR	6	3DCRT	RT area: CW and SPC	NR	None
Yu (2011) [34]	175	171	21~76	II~III	45/15	50/25	NR	7	2DRT	SPC and Axillary: 4 MV X-ray by single anterior field, and the 20 Gy of total dose was added with 5 mm bolus CW and internal mammary: 8 MV X-ray two parallel opposed tangential fields all beams supplemented by 100–200 KV X-ray	5 mm bolus to SPC and Axillary	NR
Zhang (2015) [35]	36	30	24~68	II~III	43.5/15	50/25	Toxicities	5	3DCRT	Irradiation target area: CW and SPC	NR	None
Zhao (2014) [36]	41	44	18~70	II~III	42.56/16	50/25	LRR, DM, Toxicities	6	3DCRT	CW and SPC: two parallel opposed tangential fields by using 6 MV X-ray beam	NR	None
Zhao (2016) [37]	37	35	37~59	II~III	42.56/16	50/25	OS, LRR, DM, Toxicities	7	3DCRT	RT area: CW, SPC and Axillary, excluding the Internal mammary 6 MV X-ray beam	5 mm bolus to CW	None

*OS* overall survival, *LRR* locoregional recurrence, *DFS* disease-free survival, *DM* distant metastasis, *HFRT* hypofractionated radiotherapy, *CFRT* conventional fractionated radiotherapy, *RT* radiotherapy, *NOS* Newcastle-Ottawa Scale, *2DRT* two-dimensional radiotherapy, *3DCRT* three-dimensional conformal radiotherapy, *IMRT* intensity-modulated radiotherapy, *NR* not reported, *CW* chest wall, *SPC* supraclavicular

^a^Mean age in HFRT group and CFRT group

^b^Only one stage IV case in the HFRT group

### Statistical analysis

RevMan 5.3 analysis software (Cochrane Collaboration, Copenhagen, Denmark) and STATA 14.0 (Stata Corporation, College Station, TX, USA) were used for statistical analysis. Odds ratio (OR) and 95% confidence interval (95% CI) were used for count data. Cochran’s Q test and *Ι*^*2*^ statistics were used to assess heterogeneity between studies. If heterogeneity was not present (*P* > 0.1, *I*^2^ < 50%), the fixed-effect model was adopted for analysis. Otherwise, a random-effect model was employed. The results are represented as forest maps, and potential heterogeneity was identified by sensitivity analysis. We assessed publication bias using the Egger test and funnel plots. *P* < 0.05 was considered statistically significant.

## Results

### Study selection

**Fig. 1:**
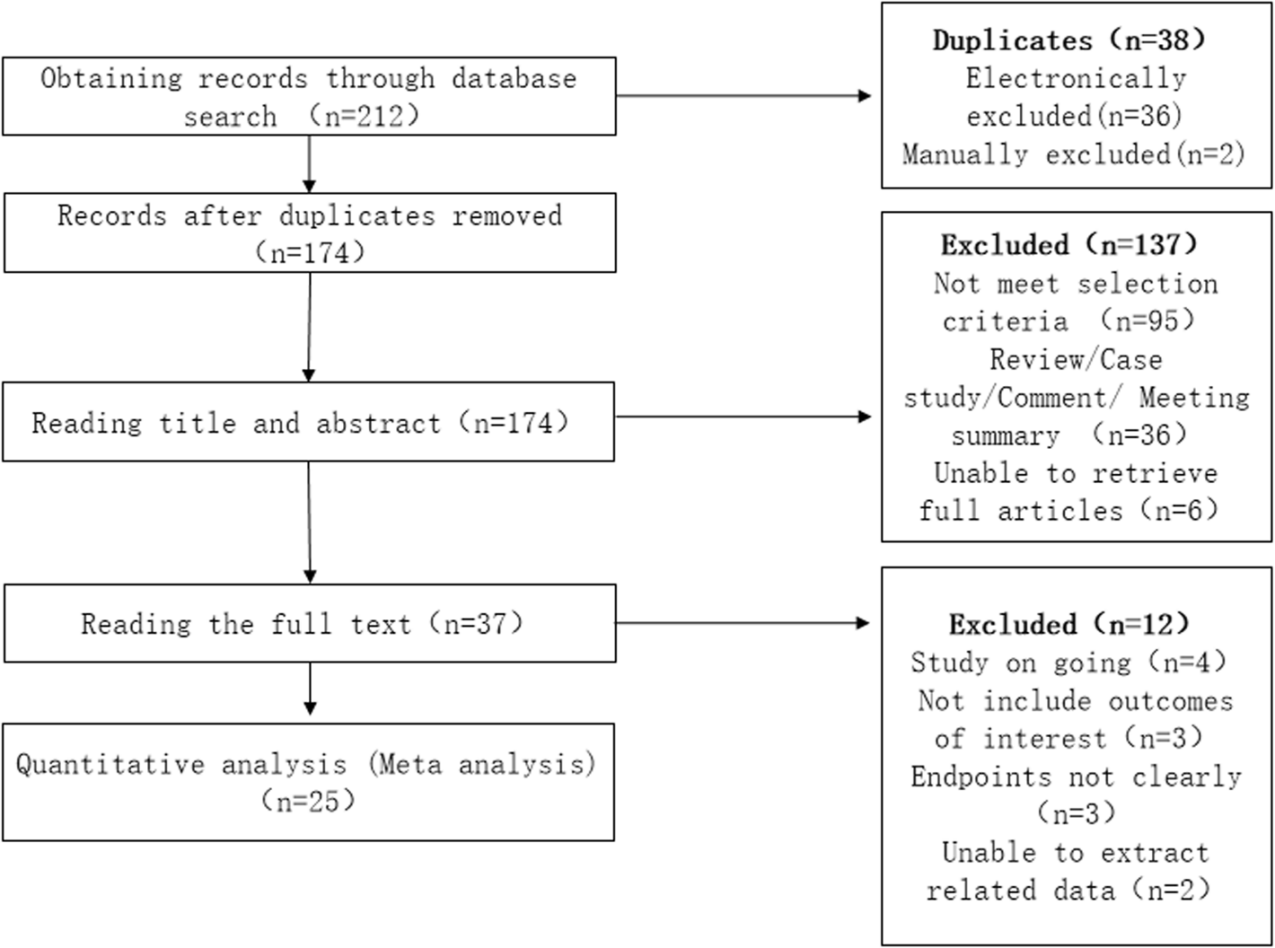
Flow chart of the study selection process

Two hundred twelve articles were initially retrieved, and after screening according to inclusion and exclusion criteria, 25 articles were entered into the systematic review [12, 14–37] (Fig. 1). Only 1 study was an RCT [12], and the rest were retrospective studies [14–37].

### Study characteristics

The characteristics of the trials are summarized in Table 1. Studies were published in 2003–2019, with a total of 3871 patients with breast cancer, including 2080 in the HFRT group and 1791 in the CFRT group. All patients underwent mastectomy with potential differences in quality between study surgeries, and none receive breast reconstruction (except the study by Yu [34] not reported). Radiation treatment area generally included the ipsilateral side chest wall and or the ipsilateral side supraclavicular area [12, 14–37]. Eight studies [18, 20, 24, 25, 28, 32, 34, 37] proposed to add the axillary fossa, axillary dome or internal mammary lymph node region if necessary. 6 MV ~ 8 MV X-ray and 6 MeV ~ 15 MeV electron beams were generally used for radiation treatment, and fourteen trials [12, 14, 15, 17–20, 25, 27–29, 31, 32, 34] used two-dimensional radiotherapy technology, with cobalt 60 being used in some patients [14, 17, 18, 27, 29]. The median age ranged from 18 to 78 years, and the TD of radiotherapy in the HFRT group ranged from 39.0 to 48.3 Gy, with a single dose of 2.3 to 3.2 Gy given over 13 to 17 fractions. The TD in the CFRT group was 50.0 Gy, with a single dose of 2.0 Gy over 25 treatments (only 1 trial [23] 48. 5 Gy in 22 fractions). Clinical characteristics between the two groups of patients included in the study, such as age, tumor stage, pathological type, estrogen and progesterone levels, HER2 status, chemotherapy regimen, etc., were not significantly different, so results were highly reliable. Patient information, including age, tumor stage, dose fraction, radiation therapy area, prescriptive method used, etc. is listed in Table 1. NOS scores are shown in Table 1.

### Meta-analysis outcomes

In the combined overall survival rate, disease-free survival rate and distant metastasis rate, there were 2 studies [19, 28], 1 study [14] and 1 study [14], respectively, that could not be submitted to meta-analysis due to no events and were not included the corresponding study outcomes for analysis.

**Fig. 2:**
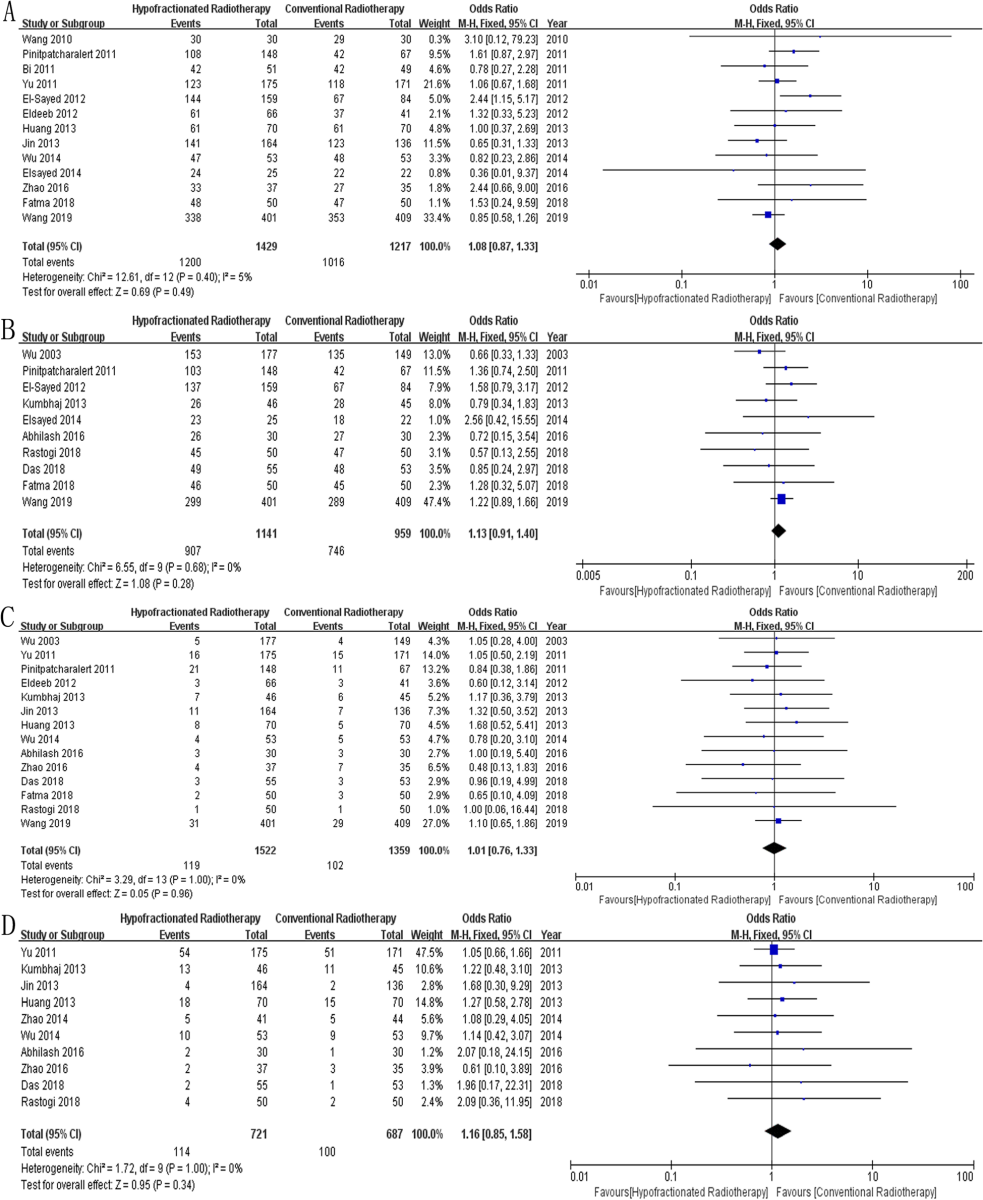
Forest plot comparing the efficacy of HFRT with that of CFRT after mastectomy in breast cancer. **a** Overall Survival, **b** Disease-Free Survival, **c** Locoregional Recurrence, **d** Distant Metastasis

**Fig. 3:**
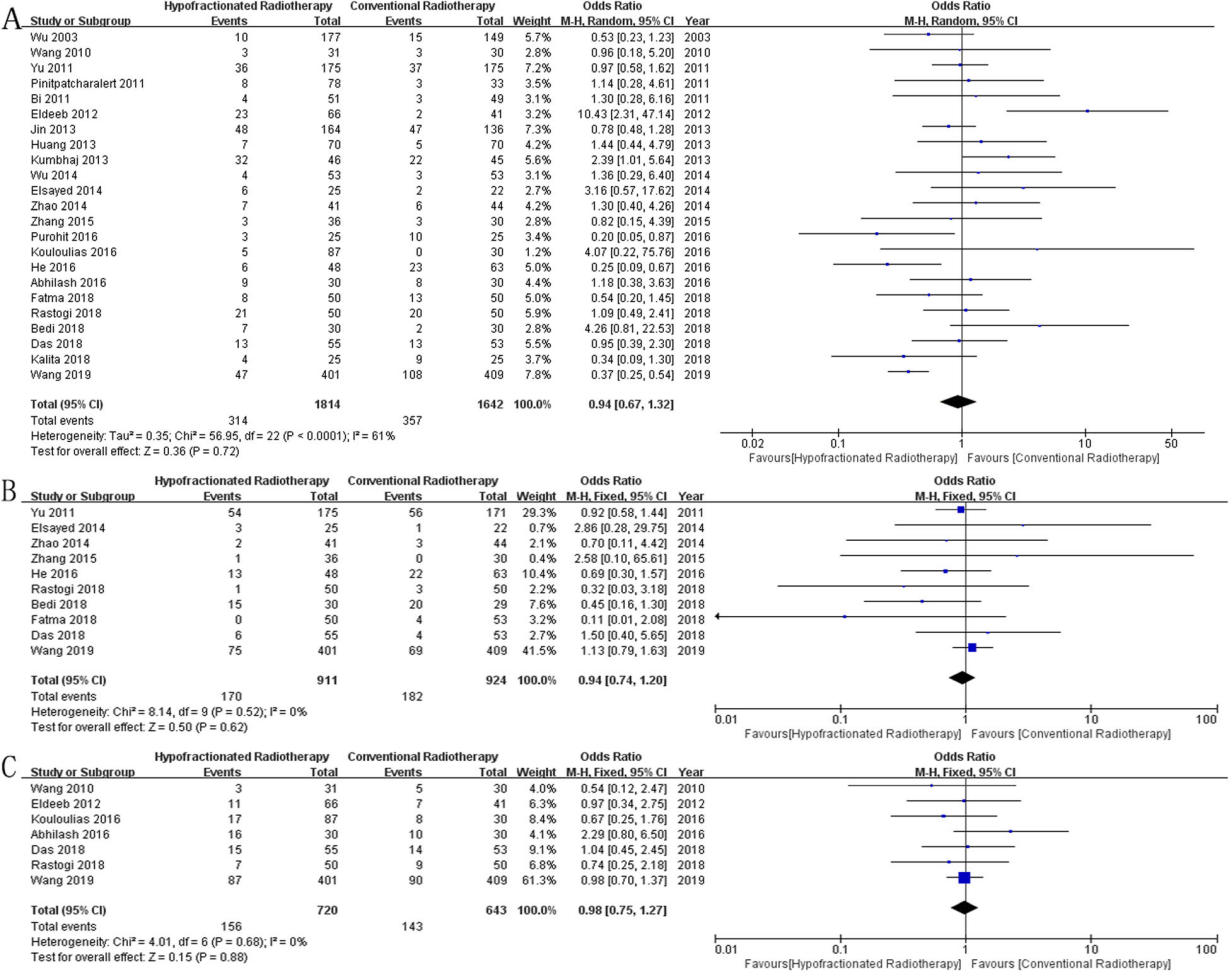
Forest plot comparing the toxicity of HFRT with that of CFRT after mastectomy in breast cancer. **a** Acute Skin Toxicity, **b** Acute Lung Toxicity, **c** Late Skin Toxicity

**Fig. 4:**
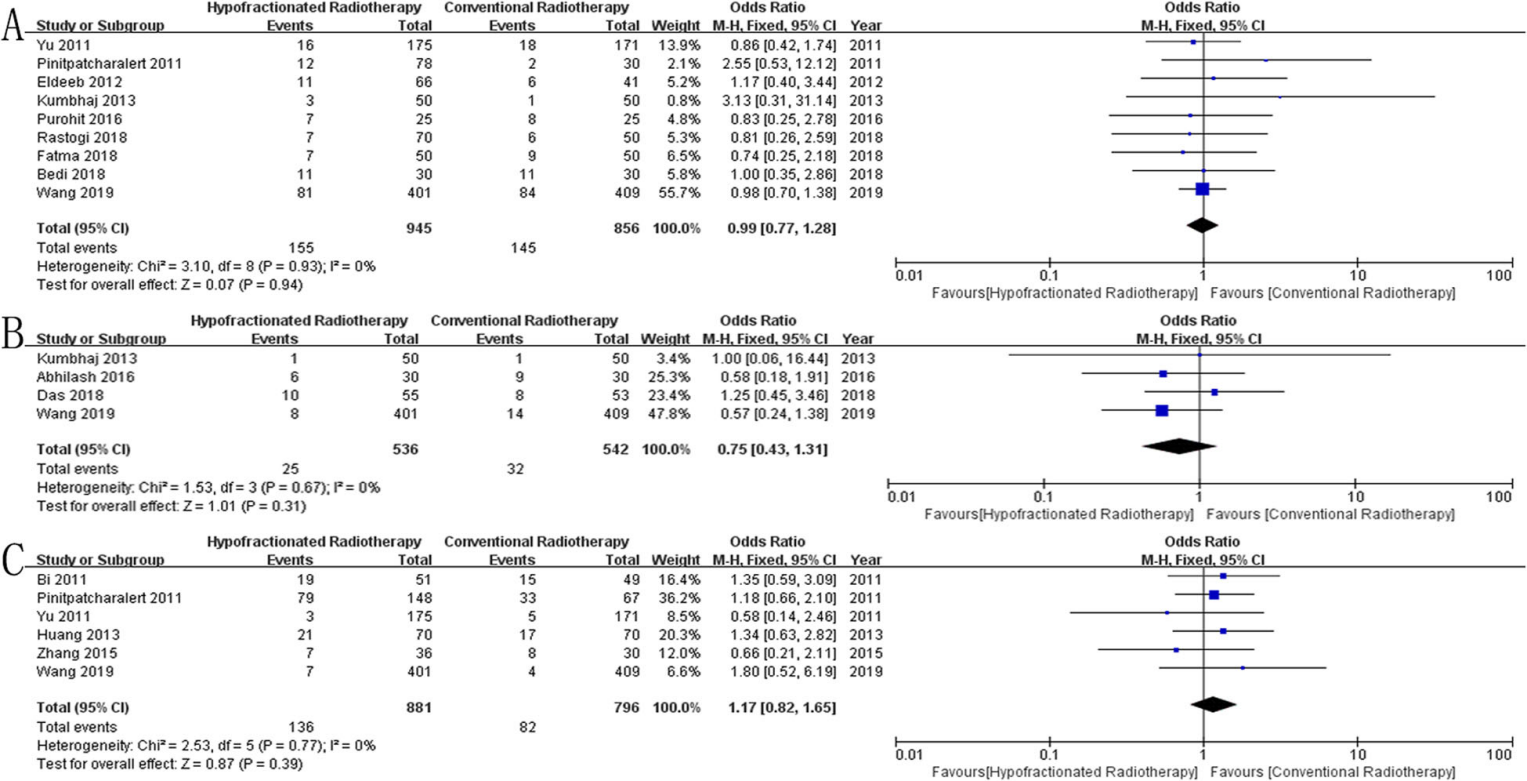
Forest plot comparing the toxicity of HFRT with that of CFRT after mastectomy in breast cancer. **a** Lymphedema, **b** Shoulder Restriction, **c** Late Cardiac Related Toxicity

1. Overall Survival: Thirteen studies [12, 15, 18–21, 23, 24, 28, 31, 33, 34, 37] reported overall survival in 2646 patients, and results showed no significant difference between the two groups (*OR* = 1.08, 95% *CI* = 0.87~1.33, *P* = 0.49, Fig. 2a).
2. Disease-Free Survival: Ten studies [12, 14, 17, 19–21, 27, 28, 30, 32] reported disease-free survival in 2100 patients, and results showed no significant difference between the two groups (*OR* = 1.13, 95% *CI* = 0.91~1.40, *P* = 0.28, Fig. 2b).
3. Locoregional Recurrence: Fourteen studies [12, 14, 17, 18, 21, 23, 24, 27, 28, 30, 32–34, 37] reported locoregional recurrence in 2881 patients, and results showed no significant difference between the two groups (*OR* = 1.01, 95% *CI* = 0.76~1.33, *P* = 0.96, Fig. 2c).
4. Distant Metastasis: Ten studies [14, 17, 23, 24, 27, 30, 33, 34, 36, 37] reported distant metastasis in 1408 patients, and results showed no significant difference between the two groups (*OR* = 1.16, 95% *CI* = 0.85~1.58, *P* = 0.34, Fig. 2d).
5. Acute Skin Toxicity: Twenty-three studies [12, 14–18, 20–36] reported acute skin toxicity in 3456 patients, and results showed no significant difference between the two groups (*OR* = 0.94, 95% *CI* = 0.67~1.32, *P* = 0.72, Fig. 3a).
6. Acute Lung Toxicity: Ten studies [12, 16, 17, 20–22, 30, 34–36] reported acute lung toxicity in 1853 patients, and results showed no significant difference between the two groups (*OR* = 0.94, 95% *CI* = 0.74~1.20, *P* = 0.62, Fig. 3b).
7. Late Skin Toxicity: Seven studies [12, 14, 17, 18, 26, 30, 31] reported late skin toxicity in 1363 patients, and results showed no significant difference between the two groups (*OR* = 0.98, 95% *CI* = 0.75~1.27, *P* = 0.88, Fig. 3c).
8. Lymphedema: Nine studies [12, 16, 18, 21, 27–30, 34] reported lymphedema in 1801 patients, and results showed no significant difference between the two groups (*OR* = 0.99, 95% *CI* = 0.77~1.28, *P* = 0.94, Fig. 4a).
9. Shoulder Restriction: Four studies [12, 14, 17, 27] reported shoulder restriction in 1078 patients, and results showed no significant difference between the two groups (*OR* = 0.75, 95% *CI* = 0.43~1.31, *P* = 0.31, Fig. 4b).
10. Late Cardiac Related Toxicity: Six studies [12, 15, 23, 28, 34, 35] reported late cardiac related toxicity in 1677 patients, and results showed no significant difference between the two groups (*OR* = 1.17, 95% *CI* = 0.82~1.65, *P* = 0.39, Fig. 4c).

### Heterogeneity analysis and publication Bias

**Fig. 5:**
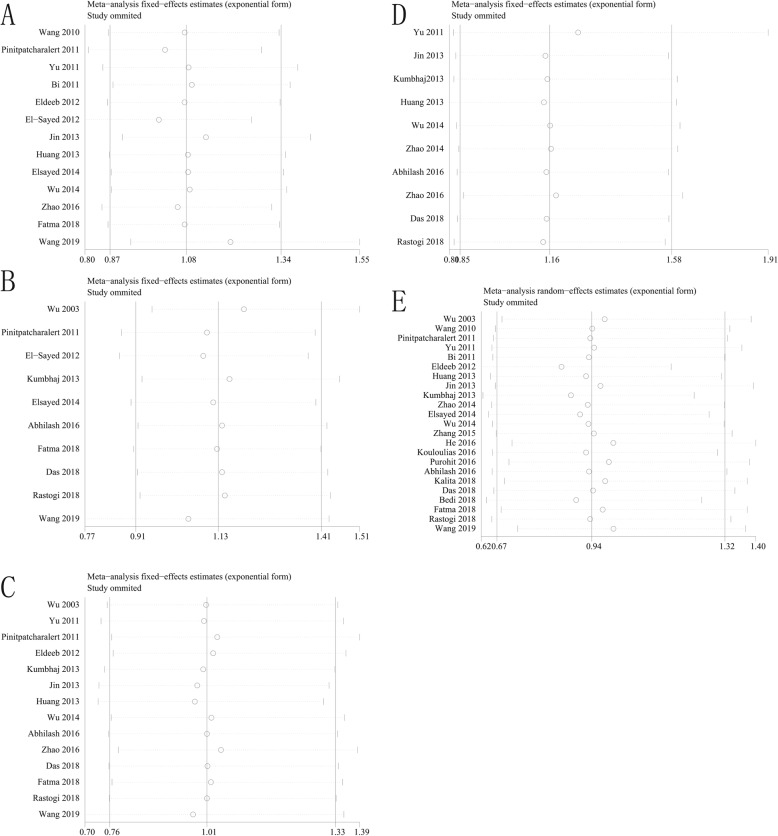
Results of sensitivity analysis. **a** Overall Survival, **b** Disease-Free Survival, **c** Locoregional Recurrence, **d** Distant Metastasis, **e** Acute Skin Toxicity

**Fig. 6:**
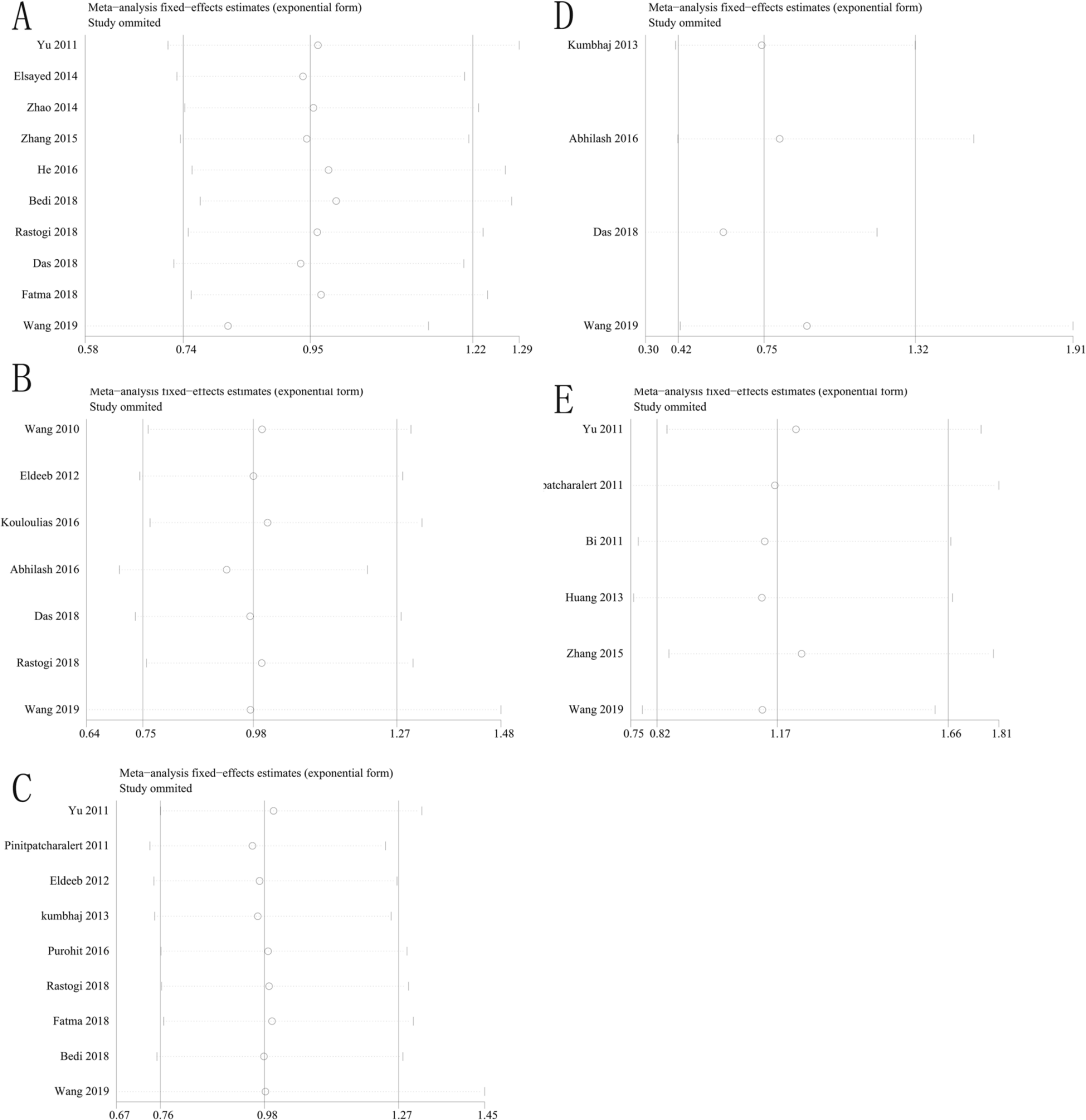
Results of sensitivity analysis. **a** Acute Lung Toxicity, **b** Late Skin Toxicity, **c** Lymphedema, **d** Shoulder Restriction, **e** Late Cardiac Related Toxicity

**Fig. 7:**
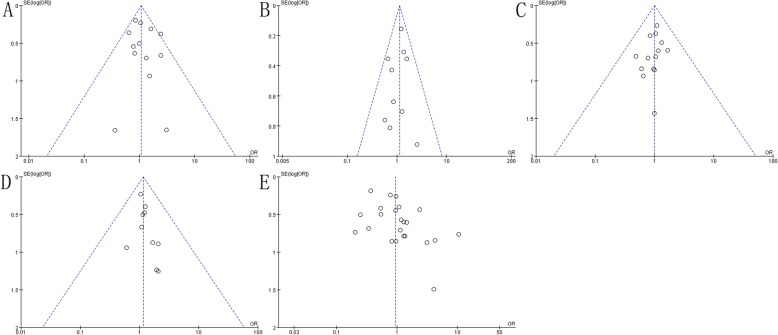
Publication biased funnel plot. **a** Overall Survival, **b** Disease-Free Survival, **c** Locoregional Recurrence, **d** Distant Metastasis, **e** Acute Skin Toxicity

**Fig. 8:**
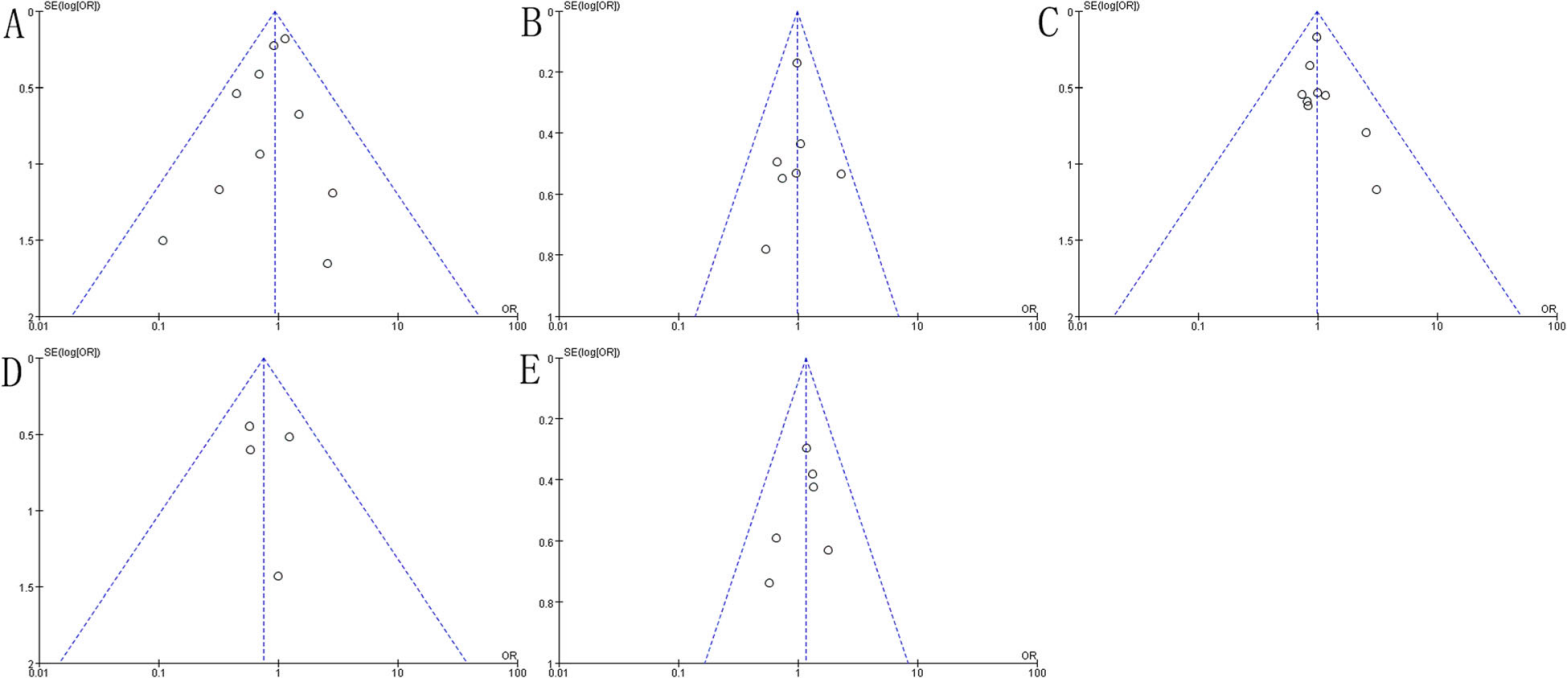
Publication biased funnel plot. **a** Acute Lung Toxicity, **b** Late Skin Toxicity, **c** Lymphedema, **d** Shoulder Restriction, **e** Late Cardiac Related Toxicity

Study outcomes included overall survival, disease-free survival, locoregional recurrence, distant metastasis, acute lung toxicity, late skin toxicity, lymphedema, shoulder restriction and late cardiac related toxicity not present heterogeneity (*P* > 0.1, *I*^*2*^ < 50%), and a fixed-effect model was adopted for analysis. The study outcome acute skin toxicity presented heterogeneity (*P* < 0.1, *I*^*2*^ > 50%), and a random-effect model was employed. Meanwhile, sensitivity analysis of this study outcome did not find any abnormal studies, indicating that our research results are more stable. Further analysis of other study outcomes using sensitivity analysis did not observe significant heterogeneity (Fig. 5, Fig. 6). Publication bias results suggest that study outcome of acute skin toxicity has a published bias (*P* < 0.05), but there was no significant bias in the remaining study outcomes (*P* > 0.05) (Table 2, Fig. 7, Fig. 8).

**Table 2:** Results of publication bias

Study Outcome	Coefficient	Standard Error	*t*	*P* > |*t*|	95% Confidence Interval
Overall Survival	0.42	0.56	0.74	0.47	−0.82~1.66
Disease-Free Survival	−0.43	0.53	−0.82	0.44	−1.64~0.78
Locoregional Recurrence	−0.39	0.33	−1.19	0.26	−1.11~0.33
Distant Metastasis	0.42	0.25	1.67	0.13	−0.16~1.00
Acute Skin Toxicity	1.57	0.64	2.45	0.02	0.24~2.89
Acute Lung Toxicity	−0.50	0.49	−1.01	0.34	−1.63~0.64
Late Skin Toxicity	−0.15	0.67	−0.23	0.83	−1.86~1.55
Lymphedema	0.41	0.38	1.07	0.32	−0.50~1.32
Shoulder Restriction	0.39	1.30	0.30	0.79	−5.19~5.98
Late Cardiac Related Toxicity	−0.89	0.97	−0.92	0.41	−3.59~1.80

## Discussion

For patients who underwent breast-conserving surgery and received whole-breast radiotherapy, long-term results from large randomized trials confirmed equivalent efficacy and safety of HFRT and CFRT. However, for patients who underwent surgical mastectomy the scarcity of high-level evidence has resulted in only a few patients having received HFRT [13], and only one randomized study has compared HFRT and CFRT in breast cancer patients who underwent mastectomy [12]. Other evidence for the clinical application of postmastectomy HFRT schedule has until now only been available from case series, retrospective studies, or subgroup analyses from the START randomized trials. Thus, we performed this meta-analysis to determine the efficacy and safety of postmastectomy HFRT schedule on outcomes in women with breast cancer. This meta-analysis indicated that HFRT and CFRT were equally effective with respect to overall survival (OS), disease-free survival (DFS), locoregional recurrence (LRR), and distant metastasis (DM) after breast mastectomy.

In recent small retrospective cohort studies, HF PMRT was shown to be effective with acceptable toxicity [38–41]. In a recent phase 2 trial [42], 67 women with clinical stage II to IIIa breast cancer who received a HF PMRT regimen of 36.6 Gy over 11 fractions to the chest wall and the draining regional lymph nodes with a scar boost of 4 fractions of 3.33 Gy revealed that after a median follow-up of 32 months, patients with isolated ipsilateral chest wall tumor recurrences were 3.0%, the 3-year estimated overall survival was 92.0% (95% *CI*, 78.9~97.1), the 3-year estimated local recurrence-free survival was 89.2% (95% *CI*, 74.8~95.6), the 3-year estimated distant recurrence-free survival was 90.3% (95% CI, 79.7~95.6), and low toxicity was reported. In *Lancet Oncology, 2019*, Shu-Lian Wang [12] reports 5-year outcomes of a randomized, non-inferiority, open-label, phase 3 trial comparing postmastectomy HFRT (43.5 Gy over 15 fractions in 3 weeks) and CFRT (50 Gy over 25 fractions in 5 weeks) directed to the chest wall and the supraclavicular and level III axillary nodal region in 820 patients with locally advanced breast cancer (at least four positive axillary lymph nodes or T3–4 tumors). All patients underwent chemotherapy, 76.5% used hormonal therapy, and the primary endpoint was 5-year locoregional recurrence. After a median follow-up of 58.5 months, there were no significant differences in the 5-year cumulative incidence of locoregional relapse (8·3% [90% *CI* 5·8~10·7] VS 8·1% [5·4~10·6]), 5-year overall survival (84%[90% *CI* 80~88] VS 86% [82~89]) and 5-year disease-free survival (74% [95% CI 70~79] VS 70% [65~76]) between the HFRT and CFRT groups. Furthermore, there was no significant difference between the two groups in the incidence of acute or late toxicities, including symptomatic radiation pneumonitis, lymphedema, ischemic heart disease, late skin toxicity, lung fibrosis or shoulder dysfunction; however, fewer patients experienced grade 3 acute skin toxicity in the HFRT group than in the CFRT group (14 [3%] of 401 patients VS 32 [8%] of 409 patients, *p* < 0.0001). No brachial plexopathy or rib fractures were observed, and frequencies of lymphedema and shoulder dysfunction were also reassuringly low, at less than 1% of grade 2 toxicity for both events. These results suggest that the HF PMRT regimen is safe and effective for patients with high-risk breast cancer, with low toxicity and high local control rate [12].

For long-term survival of breast cancer patients, the late treatment toxicities are also important. The results of this study found that there were no differences in acute skin toxicity, acute lung toxicity, late skin toxicity, lymphedema, shoulder restriction, or late cardiac related toxicity between the two groups. And we also found that no grade 2/3/4 late lung toxicity patients were observed, and the incidence of grade 1 was very low in the included studies [12, 14, 15, 23, 28, 32, 35]. Further, the randomized trial result showed that late lung toxicity may be increased in patients after HFRT (*P* = 0.08), but it was not statistically significant, which is worthy of further follow-up and research. Many studies have shown that the incidence of grade 2–4 acute skin toxicity after HF PMRT is between 10 and 25% [12, 28, 38, 39, 42], and this meta-analysis showed that the incidence of grade 2–4 acute skin toxicity in 3456 patients across 23 trials was 17.3%, which in line with reported rates. Similar results were observed in the PMRT subgroup of the UK START study (12%, 513/4451), which compared to the CFRT group, there was no significant difference in lymphedema or moderate or marked breast/shoulder/arm symptoms, etc. in patients receiving HFRT [5, 6]. Another late toxicity that should be considered is cardiac-related toxicity in patients after HFRT. Previous studies have shown that the incidence of cardiovascular events in patients with breast cancer after radiotherapy is very low, and the use of HFRT was not observed to increase that risk compared to CFRT [43–45]. The 10-year follow-up results of the UK START studies revealed that fatal cardiac events in START A and START B were 1.3 and 0.5%, respectively, while the incidence of ischemic heart disease was low (0.7%), and there was no significant difference between these two groups [5]. The proportion of patients with the late cardiac related toxicity was higher (52.1%) in the Pinitpatcharalert [28] study that the meta-analysis indicated, with 3.0% (1/67) in the CFRT group and 3.0% (3/148) in the HFRT group reporting patient deaths from cardiovascular events. This proportion is higher than in previous studies, which may be due to the small number of patients included, but there was no statistically significant difference between these two groups. The meta-analysis in our study showed no significant differences in late cardiac-related toxicity between the two groups, consistent with the above findings, indicating that HFRT does not significantly increase the risk of cardiovascular related events in breast cancer patients.

A higher dose per treatment fraction might increase the risk of toxicities in the setting of regional nodal irradiation (RNI) [46], but hypofractionated RNI was not observed to increase toxicity in one randomized clinical trial [12]. Two recent studies reported that the efficacy and safety of hypofractionated RNI were acceptable [47, 48]. One was based on UK START trials, with 864/5861 patients who experienced adjuvant lymphatic radiotherapy (LNRT) (PMRT 202/864, 23.4%) assessed using the EORTC QLQ-BR23 scale, protocol-specific questions and by physicians [47]. The long-term results from START trials suggest that appropriately dosed hypofractionated LNRT is safe, according to patient and physician-assessed arm and shoulder symptoms, a conclusion consistent with the findings for > 2.0 Gy schedules delivered to the breast/chest wall [47]. Another retrospective study reviewed 257 patients with stage IIa to IIIc breast cancer receiving hypofractionated RNI, with 80.2% patients having PMRT, 99.6% undergoing chemotherapy, 81.3% having hormonal treatment, and 25.3% having anti-HER2 targeted therapy. The median follow-up time was 64 months (range, 11 to 88 months), and the 5-year OS, DFS, locoregional recurrence (LRR)-free survival, and distant metastasis (DM)-free survival was 86.6, 84.4, 93.9 and 83.1%, respectively. During study follow-up, no acute symptomatic pneumonitis, cardiac events, brachial plexopathy or rib fractures occurred, and the incidence of grade 2–4 lymphedema was 5.8% [48]. The above findings suggest that the HFRT schedule may be acceptable in breast cancer patients who require RNI. However, prospective trials are necessary to confirm these results.

Hypofractionated radiotherapy could help to contain the costs of cancer care by mitigating financial toxicity and can be performed in most radiotherapy centers, even at small-scale hospitals. Studies have reported that the cost of using hypofractionated whole breast irradiation (WBI) in the United States is 31.7% lower than that of conventional fractionated WBI [49], and one study in Asia also indicated that the total cost of treatment for hypofractionated WBI compared to conventional fractionated WBI was reduced by about one-third [50]. It should be noted that although hypofractionated PMRT is not the same as the target area irradiated by hypofractionated WBI, the treatment technique and radiotherapy fraction are similar, and it can still shorten the treatment cycle, reduce the time of patient trips to the hospital, and save medical resources, which is more cost-effective. This issue is even more important in low- and middle-income countries.

The inclusion and exclusion criteria of this study were strict, the literature search was comprehensive, and the results are highly credible, but the following limitations do exist: 1. Quality of the included studies was unequal, and the number of included studies was limited. There may be differences in the quality of surgery between studies, and the quality of surgery could not be evaluated. There are many methods for adjuvant treatment of breast cancer, and all adjuvant treatments could not be evaluated. 2. Included studies did not provide survival data for patients of different age, tumor stage, positive lymph node numbers, pathological type, estrogen/progesterone levels, and could not be further analyzed for their impact on efficacy. However, the clinical characteristics of the two groups of patients included in the study, including age, tumor stage, pathological type, estrogen and progesterone levels, HER2 status, postoperative chemotherapy, etc., were not significantly different, so the reliability of these results was still high. 3. Most of the current studies were retrospective (only one was an RCT), and the quality of research methods is unequal. There are differences in treatment methods, radiotherapy, loss of follow-up descriptions, etc. It is difficult to extract all treatment data and then evaluate them. 4. Some trials used outdated radiotherapy techniques, and the use of hypofractionation schedules is variable. 5. Limited follow-up times in these included trials, multi-center prospective clinical trials and long-term follow-up are still needed for verification.

## Conclusions

The results of this study show that there is no statistically significant difference in efficacy or toxicity between hypofractionated radiotherapy and conventional fractionated radiotherapy after breast mastectomy. Hypofractionated radiotherapy is a safe and effective radiotherapy schedule, but the current study is still primarily retrospective and requires large-scale randomized clinical trials to confirm this conclusion along with long-term follow-up of patients who experience late toxicities.

## Data Availability

All data generated or analysed during this study are included in this published article [and its supplementary information files].

## Abbreviations

CFRT: Conventional fractionated radiotherapy
DFS: Disease-free survival
DM: Distant metastasis
HF PMRT: Hypofractionated postmastectomy radiotherapy
HFRT: Hypofractionated radiotherapy
LNRT: Lymphatic radiotherapy
LRR: Locoregional recurrence
NCCN: National Comprehensive Cancer Network
NOS: Newcastle-Ottawa scale
OR: Odds ratio
OS: Overall survival
PMRT: Postmastectomy radiotherapy
RCTs: Randomized controlled clinical trials
RNI: Regional nodal irradiation
TD: Total dose
WBI: Whole breast irradiation
